# Familial Xq27.1q28 duplication arising from a maternal interarm forward insertion of the X chromosome: a case report

**DOI:** 10.3389/fgene.2025.1514856

**Published:** 2025-11-14

**Authors:** Xiang Li, Yuan-Mei Peng, Bo-Wen Luo, Yu-Di Luo, Yun-Rong Qin, Keng Feng, De-Rong Li, Zeng-Yu Yang, Ling-Ling Zhu, Jin-Jie Pan, Ju-Jie Song, Jian Liang, Wei-Wu Liu, Guo-Sheng Deng

**Affiliations:** 1 Reproductive Medicine Center, Yulin Maternal and Child Health Care Hospital, Yulin, Guangxi, China; 2 Department of Clinical Laboratory, Yulin Red Cross Hospital, Yulin, Guangxi, China; 3 Department of Clinical Laboratory, Yulin Maternal and Child Health Care Hospital, Yulin, Guangxi, China

**Keywords:** genetic analysis, Xq27.1q28 duplication, maternal X chromosome, interarm forward insertion, recurrent adverse pregnancy, case report

## Abstract

**Objective:**

This study investigates a rare case of Xq27.1q28 duplication arising from a maternal interarm forward insertion of the X chromosome, focusing on prenatal diagnosis, family analysis, and genetic counseling for a pregnant woman with repeated adverse pregnancy outcomes.

**Methods:**

Conducted in March 2023 at Yulin Maternal and Child Health Hospital, blood samples from the proband’s mother, her husband, and her family, along with umbilical cord blood from the proband. G-banding chromosomal karyotyping and CNV-seq were performed and interpreted according to ACMG guidelines. The aim was to trace the origin of genetic variations and assess recurrence risks.

**Results:**

The grandmother had a 46,X,ins(X) (p22.1q27q28) karyotype; the proband’s mother had a 46,X,rec(X)dup (Xq)ins(X) (p22.1q27q28)dmat karyotype; and the proband had a 46,Y,rec(X)dup (Xq)ins(X) (p22.1q27q28)mat karyotype. This indicated the proband’s chromosome was inherited from the mother, originating from the grandmother. Other family members had normal karyotypes. NGS revealed a pathogenic 15.65 Mb duplication in the Xq27.1-q28 region, which was present in the proband and his mother, confirming the maternal X-chromosome insertion as the cause of the duplication.

**Conclusion:**

The grandmother’s chromosomal insertion in the Xq27.1q28 duplication in both the proband’s mother and her offspring. Given the X-linked dominant inheritance pattern, there’s a 50% recurrence risk in future offspring. Genetic counseling emphasized the importance of assisted reproductive technology (ART) for future pregnancies to mitigate the risk of recurrence.

## 1 Introduction

Chromosomal insertion, also referred to as “insertion translocation,” is a type of chromosomal structural variation characterized by three breaks in a single chromosome, where a fragment from one break is inserted into another. Based on the orientation of the inserted fragment relative to the recipient chromosome, insertions can be further classified as forward insertions or reverse insertions. These variations are typically categorized as either intrachromosomal insertions or interchromosomal insertions (Elizabeth et al., 2021; Van Hemel and Eussen, 2000).

Maternal X chromosome intercalary forward insertion (Maternal X Chromosome Intercalary Insertion) is a specific type of X chromosome structural variation. It involves a fragment of the X chromosome being inserted from one part of the chromosome into another, typically within the intercalary region of the X chromosome, with the inserted fragment maintaining its original orientation. While such insertions in the mother generally do not affect her health, they can significantly impact the chromosomal structure of the offspring. Specifically, during meiosis, this type of insertion may lead to chromosome pairing abnormalities, increasing the risk of adverse pregnancy outcomes, including miscarriage, stillbirth, and congenital defects (Wang et al., 2023).

Structural abnormalities in the X chromosome are relatively rare, particularly among women of reproductive age. A study analyzed the karyotypes of 2,936 women with reproductive issues, found that only 0.17% of cases exhibited structural rearrangements of the X chromosome, such as intercalary inversions, deletions, or translocations (Kouvidi et al., 2023).

Previous studies have shown that duplications in the Xq26.2-q27.1 and Xq26.3-q27.1 regions, which encompass the SOX3 gene, are closely associated with the development of hypopituitarism. In contrast, duplications in the Xq27.1 region are linked to intellectual disability, neural tube defects, and X-linked hypopituitarism (Du et al., 2022). Additionally, Rio et al. reported familial interstitial duplications in the Xq27.3-q28 region, which include the FMR1 gene, but exclude the MECP2 gene, which is associated with X-linked intellectual disability in males (Rio et al., 2010). Hickey et al. (2013) further described duplications in the Xq27.3-q28 region, which are associated with X-linked gonadotropin deficiency, male breast development, intellectual disability, short stature, and obesity (Hickey et al., 2013).

This study aims to explore the genetic transmission patterns, potential pathogenic mechanisms, and clinical phenotype associations of Xq27.1q28 duplications caused by maternal X chromosome intercalary forward insertion, particularly focusing on its relation to adverse pregnancy outcomes. This research will contribute to a deeper understanding of X chromosome structural variations and provide scientific evidence for clinical genetic counseling and reproductive decision-making.

## 2 Case presentation

**FIGURE 1:**
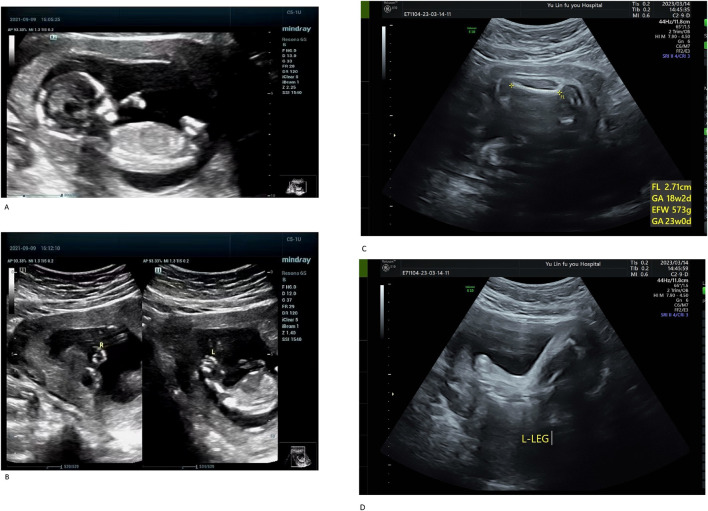
**(A)** Systemic fetal ultrasound results of the proband (III-1). **(B)** Systemic fetal ultrasound results of the proband (III-1). Ultrasound examination revealed shortened limbs, abnormal positioning of both hands (suggestive of bilateral radial aplasia), fixed posture of both lower limbs, and bilateral clubfoot. Reversed a-wave in the ductus venosus was also observed, leading to pregnancy termination **(A,B)**. **(C)** Systemic fetal ultrasound results of III-3. **(D)** Systemic fetal ultrasound results of III-3. At 26 weeks of gestation during her third pregnancy, Case III-3 underwent a level II ultrasound, which revealed shortened limbs, bilateral clubfoot, and an enlarged gallbladder **(C,D)**.

The proband (III-1) was a male fetus from the patient’s first pregnancy. At 14 weeks of gestation, prenatal ultrasound revealed significant shortening of all four limbs, abnormal hand positioning suggestive of bilateral radial aplasia, fixed lower limb posture, and bilateral clubfoot (Figures 1A,B). Additionally, reversal of the a-wave in the ductus venosus was observed. Due to multiple severe structural anomalies, the pregnancy was terminated. The fetus weighed approximately 100 g at delivery and exhibited no other discernible malformations.

The proband’s mother (II-3) is a 25-year-old woman with a height of 140 cm, an arm span of 115 cm, and a weight of 51.5 kg. Her height was notably greater than her arm span. She exhibited short limbs with bilateral elbow contractures and restricted elbow extension, reaching approximately 150° at full extension. The forearms were slightly flexed with pronation deformity. Cognitive development and intelligence were normal. The palmar ATD angle was greater than 45°. She is gravida 3, para 0, and was referred to our hospital at 26 weeks of gestation for prenatal genetic evaluation owing to a family history of fetal structural anomalies and recurrent pregnancy loss. Her second pregnancy (III-2) resulted in a biochemical pregnancy, with the fetal sex undetermined. In her third pregnancy (III-3), a prenatal ultrasound at 26 weeks revealed limb shortening, bilateral clubfoot, and gallbladder enlargement (Figures 1C,D). On 15 March 2023, cordocentesis was performed for prenatal genetic testing, followed by elective pregnancy termination.

The grandmother (I-2), a 55-year-old woman, had a height of 125 cm, an arm span of 105 cm, a sitting height of 80 cm, and a weight of 50 kg. Her height was significantly greater than her arm span. She exhibited short limbs, bilateral single transverse palmar creases, and limb deformities resembling phocomelia. The proximal limb segments were slightly shorter and smaller than normal, while the distal segments were markedly shortened. Forearm extension was limited. The palms were flexed, forming a claw-like hand appearance, with a palmar ATD angle less than 45°. The fingers appeared morphologically normal. The lower legs were short with underdeveloped gastrocnemius muscles, and foot mobility was restricted. She experienced six pregnancies. Four male fetuses (II-1, II-2, II-5, and II-7) exhibited limb shortening and died shortly after birth; none underwent genetic testing, and their gestational ages were not documented. She also had the proband’s mother (II-3) and one healthy son (II-6). Individual II-3, apart from short stature, exhibited no abnormalities in cognition, endocrine function, or skeletal structure. The height of individual Ⅱ-6 was 160 cm.

## 3 Materials and methods

To address the issue of adverse pregnancy outcomes within a family, our study aimed to identify relevant genetic factors. The proband, who was the fetus (III-3) at 26 weeks of gestation, underwent ultrasound-guided umbilical vein puncture, from which 2 mL of umbilical cord blood was extracted. DNA was extracted using a kit from Beijing Berry Harmonic Biotechnology Co., Ltd. For the proband, we utilized fetal umbilical cord blood, while peripheral blood was collected from other family members. Chromosomal karyotyping was performed by counting 30 metaphase cells and analyzing 5 karyotypes in each case. If mosaicism is suspected, the number of cells counted is doubled or even increased to 100 cells. In our analysis, no evidence of mosaicism was observed. All procedures were conducted in strict accordance with ISCN 2020 guidelines. The OMIM, DECIPHER, DGV, UCSC databases used in this study were accessed on 5 November 2021. CNV-seq was based on the human genome hg19 version, and NGS was based on the Human Genome Build (GRCh37) version.

### 3.1 CNV-seq analysis

The DNA sequence data obtained from testing were aligned with the reference genome database to determine the amount of uniquely mappable sequences on each chromosome. Based on bioinformatics analysis, the coverage depth for each chromosome was calculated and converted into an index to assess the risk of chromosomal abnormalities. The identified deletion and duplication regions, along with their associated genes, were queried in databases including OMIM, DECIPHER, DGV, and UCSC, and relevant literature was retrieved from the PubMed database. Interpretation of the results was performed according to the guidelines of the American College of Medical Genetics and Genomics (ACMG). CNV-seq analysis was based on the human genome reference version hg19. The clinical significance of CNVs was classified into five categories: pathogenic, likely pathogenic, uncertain significance, likely benign, and benign.

### 3.2 NGS analysis

MALBAC or MDA amplification products were purified, and DNA fragments of 200–2000 bp in size were recovered. CNV and SNP sequencing libraries were then constructed sequentially according to the standard operating procedures of Beijing Full Spectrum Medical Laboratory Co., Ltd. The libraries were sequenced on the NextSeq 550 platform (Illumina, United States). Sequencing data were analyzed using ChromGo software to identify pathogenic gene mutations and CNVs. NGS analysis was referenced to the Human Genome Build (GRCh37) version.

## 4 Result

### 4.1 Results of chromosome karyotyping

**FIGURE 2:**
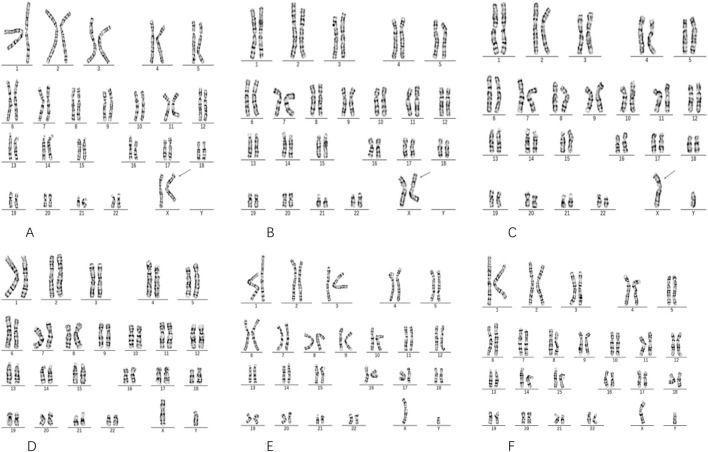
Results of Chromosome Karyotype Analysis. The pregnant woman’s mother I-2 **(A)** exhibits a karyotype of 46,X,ins(X) (p22.1q27q28). The pregnant woman II-3 **(B)** shows a karyotype of 46,X,rec(X)dup (Xq)ins(X) (p22.1q27q28)dmat. The fetus III-3 **(C)** presents a karyotype of 46,Y,rec(X)dup (Xq)ins(X) (p22.1q27q28)mat. The pregnant woman’s husband II-4 **(D)**, her brother l-6 **(E)**, and father I-1 **(F)** alI display a karyotype of 46, XY. **(A)** Shows individual Ⅰ2 (the grandmother) as a carrier of an X chromosome insertion, in which the Xq27–q28 segment is inserted into the short arm at Xp22.1. This insertion is indicated by an arrow in the image. **(B)** Displays individual Ⅱ3 (the proband’s mother), who carries a recombinant X chromosome inherited from Ⅰ2, characterized by a duplication of the Xq27–q28 region at Xp22.1. This is also marked by an arrow.

Chromosomal karyotype analysis revealed that c (I-2) carried an abnormal X chromosome with a karyotype of 46,X,ins(X) (p22.1q27q28) (Figure 2A). The proband’s mother (II-3), was identified as a recombinant carrier resulting from this insertion, with a karyotype of 46,X,rec(X)dup (Xq)ins(X) (p22.1q27q28)dmat (Figure 2B). III-3 exhibited a karyotype of 46,Y,rec(X)dup (Xq)ins(X) (p22.1q27q28)mat, consistent with a maternally inherited recombinant chromosome (Figure 2C). The karyotypes of the proband mother’s husband (II-4), her brother (II-6), and her father (I-1) were all normal male karyotypes, 46,XY (Figures 2D–F).

### 4.2 Results of NGS and CNV-seq analysis

**FIGURE 3:**
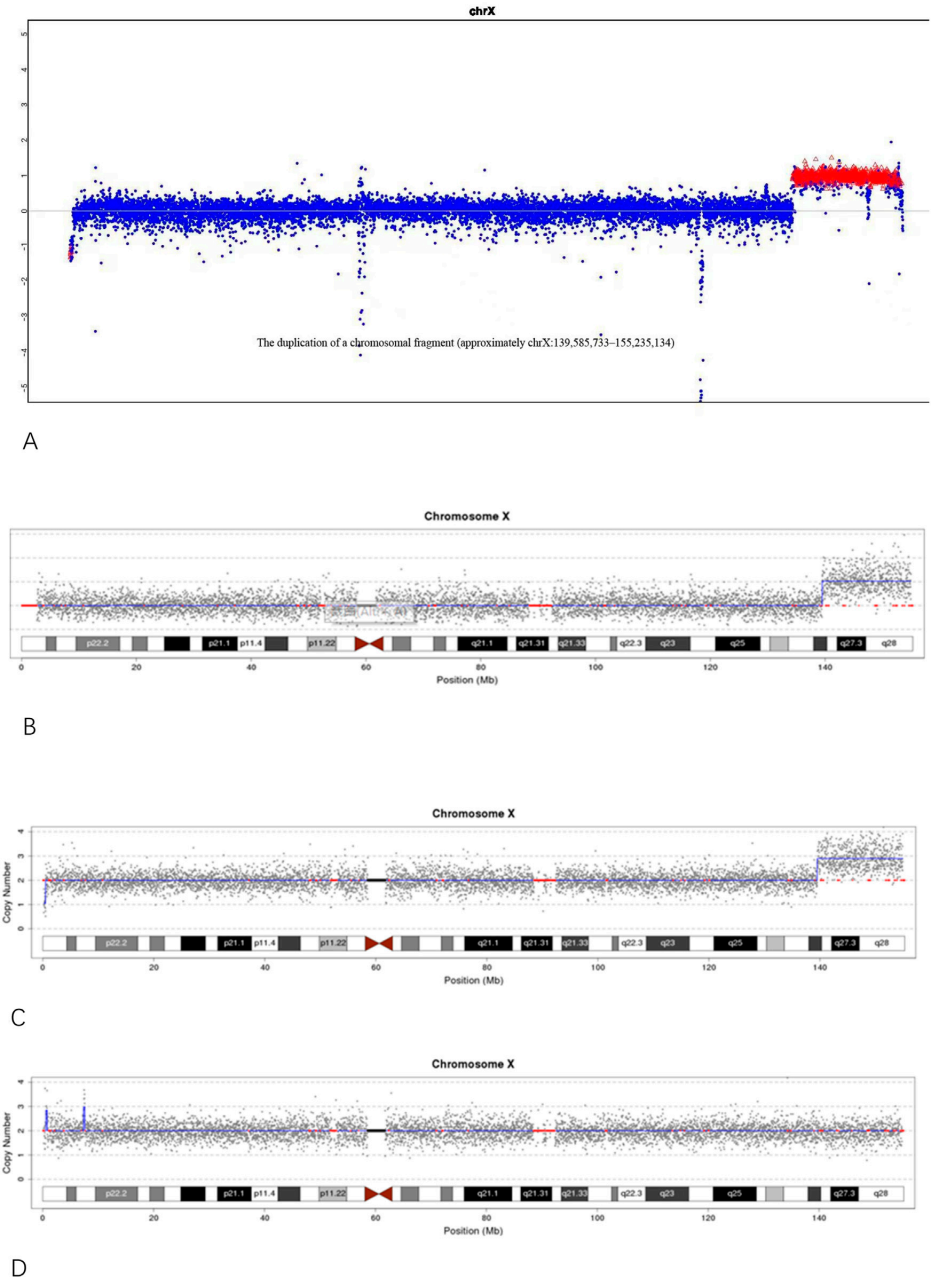
**(A)** NGS analysis of the proband (III-1) showing an approximately 15.65 Mb duplication in the Xq27.1–q28 region; **(B)** CNV-seq analysis of individual III-3 showing an approximately 15.44 Mb duplication in the Xq27.1–q28 region. **(C)** CNV-seq results of individual II-3 (the proband’s mother) showing an approximately 15.44 Mb duplication in the Xq27.1–q28 region and a 0.28 Mb deletion in the Xp22.33 region. **(D)** CNV-seq results of individual I-2 (the proband’s grandmother) showing an approximately 0.24 Mb duplication in the Xp22.33 region.

Molecular testing further clarified the nature of this chromosomal abnormality. CNV-seq analysis of individual III-3 identified a duplication of approximately 15.44 Mb in the Xq27.1–q28 region, designated as seq [hg19]dup(X) (q27.1q28)chrX:g.139500000_154940000dup (Figure 3B). This region encompasses 96 protein-coding genes and is classified as a pathogenic variant based on a ClinGen dosage sensitivity score of ≥0.99. A similar duplication of approximately 15.65 Mb in the same region was previously detected by NGS in the proband (III-1, a stillborn male) (Figure 3A), indicating that both fetuses inherited an identical duplicated segment derived from the same maternal inserted chromosome. The proband’s mother (II-3) also carried this duplication, confirming the structural variant as a stable, maternally transmitted insertion originating from individual I-2. Further genetic characterization showed that individual II-3 (the proband’s mother) had a 15.44 Mb duplication in the Xq27.1–q28 region, corresponding to seq [hg19]dup(X) (q27.1q28)chrX: g.139500000_154940000dup, and a 0.28 Mb deletion at Xp22.33, designated as seq [hg19]del(X) (p22.33p22.33)chrX: g.160000_440000del (Figure 3C). In contrast, individual I-2 (the proband’s grandmother) exhibited a 0.24 Mb duplication at Xp22.33, designated as seq [hg19]dup(X) (p22.33p22.33)chrX: g.560000_800000dup (Figure 3D). These findings suggest that the complex rearrangement in II-3 may have arisen from an inter-arm insertional event on the X chromosome transmitted from I-2.

### 4.3 Pedigree analysis and inheritance pattern

**FIGURE 4:**
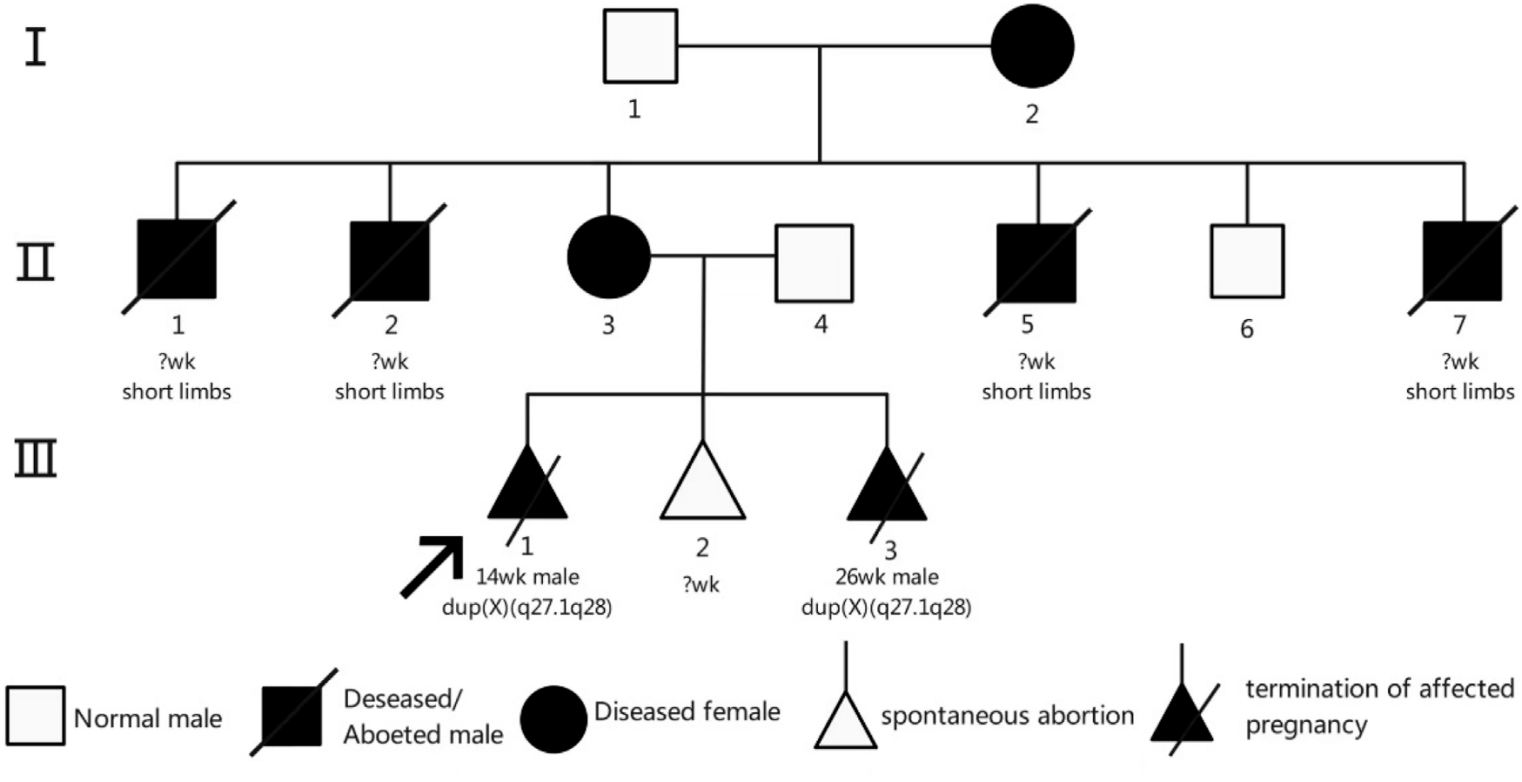
Results of the family investigation for the proband (III-1).

Pedigree analysis revealed a typical X-chromosome inheritance pattern in this family (Figure 4). Individual II-3, apart from short stature, exhibited no abnormalities in cognition, endocrine function, or skeletal structure. In contrast, individual I-2, in addition to short stature, had deformities of the hands and feet, was unable to place the heels on the ground when walking, and had one eye that could not open. Neither individual displayed intellectual disability. The female carriers I-2 and II-3 presented with varying degrees of clinical manifestations, including short stature (125 cm and 140 cm, respectively) and a history of adverse pregnancies. I-2 had six children, four of whom were male (II-1, II-2, II-5, and II-7) and presented with short limbs, dying shortly after birth; no chromosomal testing was performed in these cases. II-3 was a confirmed carrier, whereas II-6 had a normal phenotype. III-1 underwent pregnancy termination due to the absence of bilateral radius bones and inward-turned feet. III-3 carried the same Xq duplication. The height of II-6 was approximately 160 cm.

### 4.4 Genetic counseling

Based on the integration of chromosomal karyotyping, CNV-seq results, and pedigree information, this study identified an interstitial insertion of the X chromosome originating from I-2, which resulted in a duplication of the Xq27.1 - q28 region. This duplication has been stably inherited through the maternal line to the third generation and is closely associated with multiple adverse pregnancy outcomes. The duplicated segment spans 96 protein-coding genes and has a pathogenicity score of ≥0.99 according to the ClinGen database, classifying it as a pathogenic variant. Due to variability in duplication size, insertion site, and patterns of X chromosome inactivation (e.g., random, skewed, or gene escape), female carriers may exhibit markedly variable phenotypes. Given that individual II-3 has experienced three abnormal pregnancies and the structural variant carries a 50% recurrence risk consistent with X-linked inheritance, genetic counseling was provided to explain the associated reproductive risks and available options, including the possibility of pregnancy continuation or termination. Preimplantation genetic testing (PGT) in conjunction with assisted reproductive technologies was advised for future pregnancies to minimize the risk of recurrence and ensure the birth of a healthy child.

## 5 Discussion

This study reports a familial duplication of the Xq27.1 - q28 region caused by an interstitial insertion on the X chromosome originating from I-2. This structural variant was transmitted through a recombinant X chromosome in II-3 to III-1 and III-3, both of whom exhibited severe structural abnormalities or intrauterine demise during the fetal period. Chromosomal karyotyping and CNV-seq analyses revealed that the duplicated region spans approximately 15.44–15.65 Mb and involves 96 protein-coding genes, including the Xq28 recurrent region clinically classified as pathogenic in the ClinGen database which encompasses key genes such as MECP2, GDI1, and RAB39B. In accordance with the 2020 International System for Human Cytogenomic Nomenclature (ISCN), the structural features of the maternally derived recombinant X chromosome were described using terms such as rec(X), dup (Xq), and dmat/mat.

**FIGURE 5:**
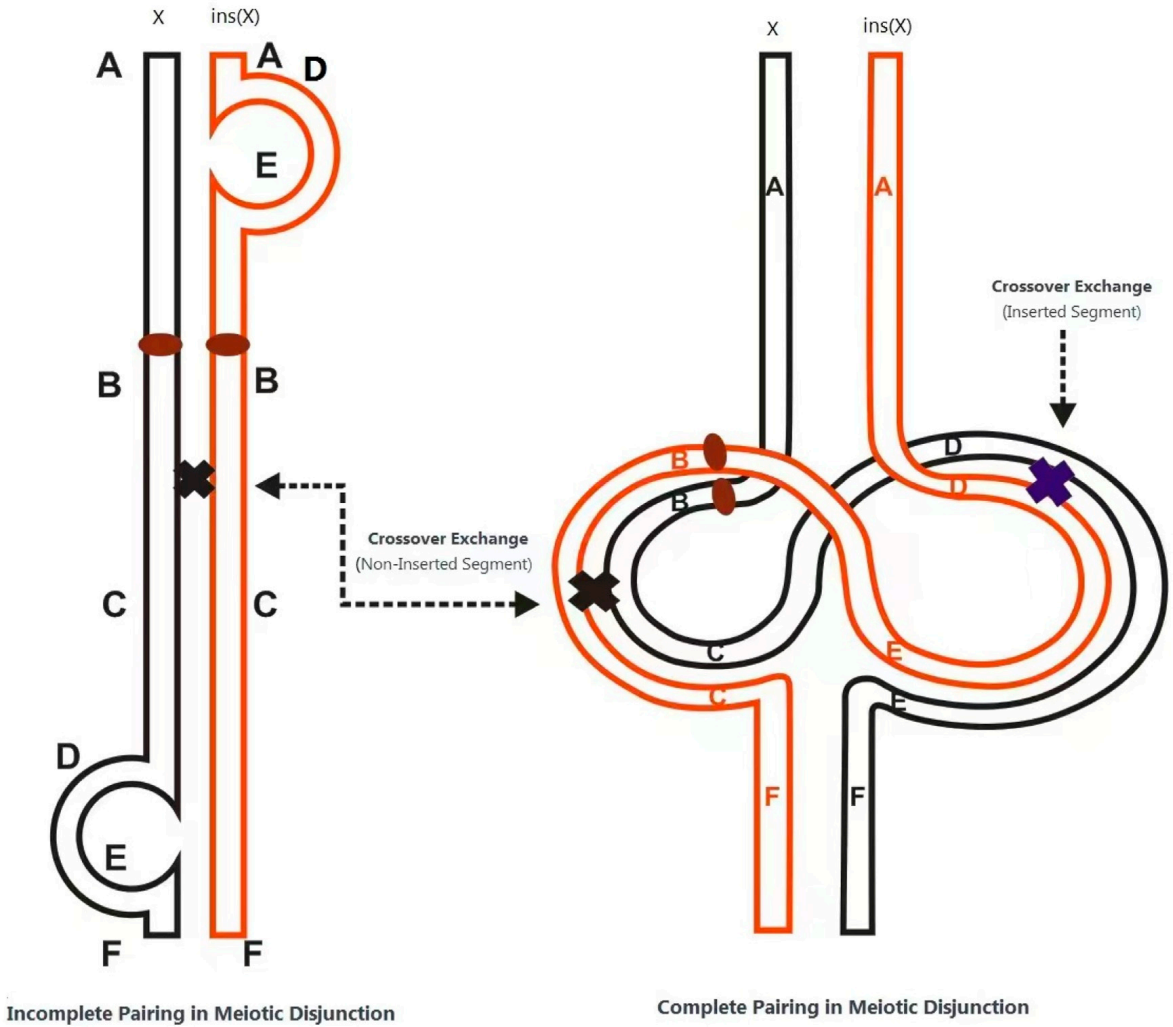
Mechanism of gamete formation in carriers of X-Chromosome arm intercalary Insertions.

The structural rearrangement observed in this family follows an X-linked dominant inheritance pattern. Female carriers typically exhibit no obvious clinical features or only mild neuropsychiatric symptoms such as anxiety, depression, or autism-like behaviors. In contrast, male carriers may exhibit severe phenotypes or embryonic lethality due to a lack of dosage compensation. The underlying pathogenic mechanism is hypothesized to involve the formation of two recombination loops during meiosis in the insertion carrier, resulting in unbalanced recombinant chromosomes through unequal crossing over. In cases of direct (non-inverted) insertion, acentric fragments or dicentric chromosomes may be produced; inversions can generate gametes with duplications or deletions. When such gametes combine with normal ones, the resulting zygotes may exhibit partial trisomy or monosomy (Figure 5). This mechanism explains the high rate of unbalanced gametes associated with such structural variants (Chan et al., 2018).

The duplicated region in this case includes the SOX3 gene located at Xq27.1, which has been previously implicated in neural tube defects and pituitary malformations. For instance, Rio et al.described a family with a small Xq27.3 - q28 duplication including FMR1 but excluding MECP2, in which affected individuals presented with intellectual disability, short stature, hypogonadism, and facial anomalies (Rio et al., 2010). Similarly, Hickey et al. reported an 8.05 Mb duplication involving FMR1 associated with obesity, gynecomastia, cognitive impairment, and pubertal delay (Hickey et al., 2013). These findings suggest that duplications in the Xq27.1 -q28 region may lead to complex phenotypes via the combined overexpression of multiple dosage-sensitive genes such as SOX3, FMR1, and MECP2.

In addition, Higgins et al. reported a fetus detected via non-invasive prenatal screening (NIPS) to carry a duplication of Xq27.1 - q28 along with a deletion at Xp22.3, presenting with congenital anomalies such as gallbladder enlargement and hydrocephalus. This further highlights the value of high-throughput genomic technologies in prenatal genetic assessment (Higgins et al., 2011).

This study extends the literature on Xq27.1–q28 duplications by presenting detailed description of a familial duplication caused by X-chromosome insertion and Its adverse effects on male fetuses. It enhances the understanding of pathogenic mechanisms underlying structural X-chromosome anomalies. Importantly, individual II-3 experienced three consecutive abnormal pregnancies, with two male fetuses confirmed to carry this CNV, indicating a high degree of pathogenicity and recurrence risk.

For this family, preimplantation genetic testing (PGT) can be applied to reduce the recurrence risk and improve reproductive outcomes. Specifically, PGT-SR (for structural rearrangements) should be performed to distinguish embryos with normal or balanced karyotypes from those carrying the duplication or unbalanced chromosomal rearrangements. In addition, PGT-A (for aneuploidy screening) can be combined to identify euploid embryos, thereby further improving implantation and live birth rates. If the chromosomal breakpoints have been precisely defined, a customized probe design based on next-generation sequencing (NGS) or SNP microarray can be used to increase the accuracy of detecting the duplication or insertional event. Alternatively, linkage analysis with polymorphic markers flanking the duplicated region can help trace the transmission pattern of the rearranged chromosome. Through this combined PGT-SR and PGT-A strategy, embryos free from the Xq27.1–q28 duplication can be selected for transfer, substantially reducing the risk of recurrent abnormal pregnancies and improving the likelihood of a successful live birth.

## 6 Conclusion

In this study, we conducted an extensive chromosomal karyotype analysis and CNV-seq testing on a family lineage affected by maternal intercalary insertion on the X-chromosome arm, resulting in a duplication in the Xq27.1q28 region. We investigated the correlation between the clinical phenotype of the proband and their genotype, thereby augmenting the available data on Xq27.1q28 duplication variants and offering valuable insights for the diagnosis and comprehension of analogous cases. Considering the Xq27.1q28 duplication variant carried by the pregnant woman and its X-linked inheritance traits, there is a substantial 50% recurrence risk for her offspring. Consequently, we recommend employing assisted reproduction techniques such as *in vitro* fertilization and preimplantation genetic testing (PGT) to mitigate the likelihood of similar genetic variations in future pregnancies.

## Data Availability

The original contributions presented in the study are included in the article/supplementary material, further inquiries can be directed to the corresponding authors.
